# Perceptions of Animal Welfare With a Special Focus on Turkeys

**DOI:** 10.3389/fvets.2019.00413

**Published:** 2019-11-21

**Authors:** Courtney Bir, Melissa Davis, Nicole Widmar, Stacy Zuelly, Marisa Erasmus

**Affiliations:** ^1^Department of Agricultural Economics, Purdue University, West Lafayette, IN, United States; ^2^Department of Animal Sciences, Purdue University, West Lafayette, IN, United States

**Keywords:** animal welfare, demographics, food labels, meat consumption, public perceptions, turkey knowledge

## Abstract

Meat consumption and public concern for farm animal welfare are increasing, despite limited public understanding of agriculture and animal welfare. Turkey is important in U.S. holiday meal traditions and turkey meat is a frequently consumed processed product (i.e., lunchmeat). However, little is known about public perceptions and knowledge of commercial turkeys. An online survey was administered to 1,695 respondents in November 2018 to examine U.S. (1) demographic factors affecting meat consumption, selection of labeled meat products, and concern for animal welfare, (2) public knowledge of turkeys, and (3) concerns regarding the welfare of turkeys and other species. A total of 95% of respondents consumed meat and 10% hunted for some of the meat they consumed. Meat consumption frequency depended on region of residence, income level, gender, age, and whether respondents hunted. Of the meat consumers, 86% purchased turkey products. More meat consumers looked for the USDA organic label (39%) and the Non Genetically Modified Organism (GMO) project label (38%) than animal-welfare food labels (14%) when buying meat products. More pet owners (39%) than non-pet owners (25%) looked for animal welfare food labels. Being a pet owner increased the probability of being concerned about farm animal welfare. Concern for the commercial turkey was similar to concern for other farm animal species; self-reported knowledge of turkey production was low (mean score 2.64; scale of 1 to 7, 7 = highest). Turkey welfare concerns (mean score; rank from 1 to 5; 5 = least concerning) included poor nutrition (2.471) and illness (2.508), followed by housing (2.732), hot or cold weather (3.308) and transportation (3.981). Turkey welfare attributes that respondents cared the most about (mean score; scale of 1–5, 5 = cared the least) included space to move around (2.366), followed by veterinary health and wellness (2.680), ability to perform natural behavior (2.812), no feather loss or visible injuries (3.304), and decreased aggression (3.837). Demographic factors are important determinants of meat consumption and animal welfare concern. Public knowledge of turkey production is limited, despite a large percentage of the population purchasing turkey products.

## Introduction

With the projected increase in the world's population to 9.1 billion by 2050, food production will need to increase by 70% (1), all the while being sustainable and taking animal welfare into consideration. Poultry production will comprise the largest proportion of world meat production by 2050 compared to any other type of meat (2). Chicken meat is the most consumed poultry meat in the U.S., but consumption of turkey meat has also increased in the past few decades and turkeys are an important part of U.S. holiday traditions. In 2018, U.S. broiler chicken production totaled over $31.746 billion in production value (over 9 billion animals produced) and U.S. turkey production totaled $3.875 billion in value (over 244 million animals produced) (3). Most of the world's live turkey production occurs in North America (4, 5). The increase in turkey and meat production has been paralleled by an increase in public interest in animal welfare, which has partly driven changes to legislation concerning the housing and management of farm animals (e.g., Proposition 2 of 2008 in California and Public Act 117 of 2009 in Michigan), despite few members of the public being familiar with agriculture and agricultural practices (6). In addition to the increase in legislative changes, the number of animal welfare certification programs that enable animal products to be labeled as “welfare-friendly” has also increased. However, it is unclear to what extent U.S. meat consumers seek out these labeled products. Furthermore, there is little information available regarding public perceptions and knowledge of turkeys and turkey welfare. As meat production and public interest in animal welfare continue to increase, public perceptions will continue to influence agricultural practices. Therefore, it is important to understand factors influencing meat consumption and public perceptions of animal welfare. This study examined U.S. (1) demographic factors affecting meat consumption, selection of labeled meat products and concern for animal welfare, (2) public knowledge of turkeys, and (3) concerns regarding the welfare of turkeys and other animal species.

## Materials and Methods

Using an online survey tool Qualtrics, the survey instrument was administered November 12-19, 2018 to accumulate demographic information, meat consumption information, and perceptions of animal welfare with a special focus on turkeys and knowledge of turkey production. With the special focus on perceptions of turkey welfare, the timing of this particular survey instrument administration was important due to the prominence of turkey consumption during the American Thanksgiving holiday. The survey was administered to end prior to Thanksgiving Day in 2018, which occurred on November 22nd, yet occur close enough to the holiday that turkeys were top-of-mind. One-thousand-six-hundred-and ninety-five respondents completed the survey instrument. Survey respondents were contacted through a company that hosts a large opt-in panel database, Lightspeed GMI. Respondents were required to be 18 years of age or older to participate. Using quotas in Qualtrics, the sample was targeted to be representative of the U.S. population in terms of gender, income, education, and geographical region of residence (7). Regions of residence were defined as in the Census Bureau Regions and Divisions^1^. The survey instrument was designed to collect information regarding general meat consumption, knowledge of turkey production, beliefs regarding food animal welfare, and specific beliefs regarding turkey welfare including housing. Frequencies were calculated for categorical variables and means were calculated for the continuous variables. The test of proportions was conducted to determine the statistical representativeness of the survey respondents by comparing percentages of demographic groups from the sample to the targeted population, the U.S. Census.

### Survey Instrument, Statistical Testing, and Models

To better understand food consumption and grocery shopping, respondents were asked questions such as whether they were the primary shopper, meat consumption of the household, and hunting behavior. The survey was designed so that respondents who did not eat meat were not asked specific meat eating questions. The test of proportions was used to compare demographic variables within consumers of nine different food species: poultry, pork, beef cattle, lamb, fish, shellfish, buffalo or bison, game species, and exotics. Respondents who indicated they consumed or someone in their household consumed meat were asked to identify from pictures of meat labels the labels they look for when buying meat products. Multiple selections were permitted.

Respondent ability to identify turkeys was evaluated by providing pictures of species of birds that are farmed or hunted, namely: a chicken, a duck, a wild turkey, and a commercial turkey, and asking respondents to identify the turkey(s). This knowledge was further tested through identification of pictures of wild and commercial turkeys of both sexes. Two questions were developed to test respondent's general knowledge of turkeys, “what is the average weight of a mature hen (female turkey)” and “how long do turkey eggs incubate before they hatch.” Respondents were also asked to indicate on a Likert scale their self-reported knowledge of turkeys. Correlations between those who correctly answered the questions and self-reported knowledge scores were determined.

A series of animal welfare related questions were presented to respondents including “can animals feel pain” and specifically “can turkeys feel pain.” The correlation between respondent's self-reported knowledge of turkeys and their response to whether turkeys can feel pain was determined. Respondents were also asked to rank their level of concern for farmed turkeys, turkeys in backyard flocks, and wild turkeys. Six conditions turkeys may face and that influence turkey welfare [reviewed in (8)] including housing type, transportation, hot or cold weather, illness, and poor nutrition were presented to respondents, and they were asked to rate their level of concern for each condition on a Likert scale. Additionally, respondents were asked to rate their level of care on a Likert scale regarding five turkey welfare attributes: space to move around, animal health and wellness, ability to perform natural behavior, no feather loss or visible injuries, and decreased aggression between animals. The mean responses for this set of questions were statistically tested using a *t*-test. Respondents were also provided three different turkey housing pictures and were asked to rank the pictures in order from best condition to worst condition. The mean responses for each of the sets of Likert scale questions, and the ranking question were statistically tested using *t*-tests.

Seven pictures of animals: a crab, a commercial turkey, a chicken, a beef cow, and a dairy cow were presented to respondents. Respondents were asked to move the picture of at least three of the animals into buckets labeled “concerned about this animal's welfare,” “neutral about this animal's welfare,” and “not concerned about this animal's welfare.” The percentage of respondents who moved each animal to the different buckets were tested across the buckets for each animal, and across the animals within each bucket using the test of proportions. To determine the relationship between moving an animal picture to concerned about this animal's welfare and demographics, seven logit models were employed. The logit models were used to estimate the probability a respondent would move the picture of the animal to the concerned bucket. Moving the picture of the crab, commercial turkey, wild turkey, chicken, commercial hog, beef cow, and dairy cow to the concerned bucket each served as an independent variable for a logit model. For comparison purposes, the same demographic variables were used in each variable with one exception. For each animal, whether the respondent was a frequent consumer of that particular animal differed between the models. Being a frequent consumer of shellfish was used in the crab model, being a frequent consumer of poultry was used in the commercial turkey, wild turkey, and chicken models, being a frequent consumer of beef was used in the beef cow and dairy cow models, and being a frequent consumer of pork was used in the pig model. The coefficients of logit models are not directly interpretable, so marginal effects were calculated. The utility *(V*_*nj*_*)* of moving an animal picture to the concerned bucket takes the form:

(1) $$\begin{array}{l} V_{nj}=\beta^{\prime }x_{nj}+e_{nj} \end{array}$$

where *x*_*nj*_ is the vector of observed variables that relate to the choice *j* for respondent *n* and *e*_*nj*_ is the unobserved error term (9). Assuming the error term is an independently and identically distributed extreme value, following (9) the logit probability *(P*_*ni*_*)* for attribute *i* and respondent *n* becomes:

(2) $$\begin{array}{l} P_{ni}=\frac{e^{\beta^{\prime }x_{ni}}}{\sum_{j}e^{\beta^{\prime }x_{nj}}} \end{array}$$

Two videos were shown to respondents. The videos were chosen to show two groups of commercials turkeys behaving differently in the same type of enclosure. One of the videos showed some of the turkeys behaving aggressively toward one another including behaviors such as pecking, chasing, and threatening displays. On the other video, turkeys were not displaying aggression. After viewing the videos, respondents were asked to select from a list of descriptive words what emotions the video elicited in them. Respondents had to choose at least one word. The list included the option none, so respondents could select none only if none of the words provided were elicited by the videos. The words were chosen by a panel of experts including experts in animal behavior, consumer preferences, turkey production, and agricultural extension. The percentages of respondents who chose each word were statistically compared between the two videos using the test of proportions. Furthermore, the percentage of select demographics that selected specific words for each video were statistically compared using the test of proportions.

## Results

### Demographics and Meat Consumption

The demographics of respondents closely matched that of the U.S. Census with a few statistically different exceptions (Table 1). There was a lower percentage of respondents who were aged 18–24 (8%), had incomes of $100,000 and higher (18%), did not graduate from high school (3%), and from the Midwest (22%) when compared to the U.S. Census: 13, 26, 13, and 38%, respectively. Higher percentages of respondents had attended college no degree earned (24%), attended college Associate's or Bachelor's degree earned (34%), attended college graduate or professional degree earned (14%), and from the South (36%) when compared to the U.S. Census: 21, 27, 12, and 21%, respectively. Sixty-seven percent of respondents indicated they had at least one pet.

**Table 1:** Demographics (*N* = 1,695).

**Demographic Variable**	**Percentage of Respondents**	**U.S. Census**
Gender		
Male	45%^+^	49%
Age		
18–24	8%^++^	13%
25–34	17%	18%
35–44	17%	16%
45–54	19%	17%
55–65	18%	17%
65 ^+^	21%^+^	19%
Income		
$0–$24,999	25%^+^	22%
$25,000–$49,999	25%^+^	23%
$50,000–$74,999	19%	17%
$75,000–$99,999	13%	12%
$100,000 and higher	18%^++^	26%
Education		
Did not graduate from high school	3%^++^	13%
Graduated from high school, Did not attend college	25%^+^	28%
Attended College, No Degree earned	24%^++^	21%
Attended College, Associates or Bachelor's Degree earned	34%^++^	27%
Attended College, Graduate or Professional Degree earned	14%^+^	12%
Region		
Northeast	19%	18%
South	36%^++^	21%
Midwest	22%^++^	38%
West	23%	24%
Respondent has a pet	67%	
Primary shopper of household	88%	
Meat consumption		
Respondent consumes meat	95%	
Respondent doesn't consume meat, but someone in their household does	2%	
No one in their household consumes meat	3%	

+ Percentage of respondents is statistically different from the percentage of the U.S. Census at the 0.05 level.

++ *Percentage of respondents is statistically different than the percentage of the U.S. Census at the <0.001 level*.

Eighty-eight percent of respondents indicated they were the primary shopper of the household (Table 1). Ninety-five percent of respondents consumed meat, 2% did not consume meat but someone in their household did, and 3% had no-one in their household who consumed meat. Of the respondents that consumed meat or had someone in their household who consumed meat (*n* = 1,649), 6% raised at least some of the meat they consumed, 10% hunted for at least some of the meat they consumed, and 88% did neither. For use in later analysis, a variable was created that indicated whether the respondent either hunted for or raised at least some of the meat they consumed. Fourteen percent of all respondents either raised or hunted for at least some of the meat they consumed. Eighty-six percent of respondents whose household consumed meat indicated they purchased turkey products, 12% indicated they did not purchase turkey products, and 2% indicated they did not know (*N* = 1,649).

Results of respondent's demographics and frequency of consuming 9 species of meat are presented in Table 2 (*n* = 1,649). Poultry, beef, and pork were consumed frequently by respondents. To further examine meat consumption, analyses were conducted using the percentage of respondents in different demographics categories that consumed meat at least weekly (note for this analysis it was assumed that those who did not consume meat did not consume any species; percentages used for the analysis of frequent consumption are out of the full sample of *n* = 1,695). Many differences due to demographic factors were found.

**Table 2:** Percentages of respondents who consume specific species.

	**Poultry**	**Pork**	**Beef Cattle**	**Lamb**	**Fish**	**Shellfish**	**Buffalo/Bison**	**Game Species**	**Exotics**
**Frequency of consumption** ***n*** **=** **1,649**									
Daily	5	3	4	2	2	2	2	2	2
4–6 times a week	17	7	10	3	3	3	3	2	2
2–3 times a week	40	18	34	3	3	5	2	4	3
Once a week	24	33	28	4	4	13	3	4	2
Monthly	9	28	14	20	20	34	9	12	7
Never	5	11	10	68	68	43	81	76	84
**Percentage of respondents of each demographic who consume the species at least weekly** ***n*** **=** **1,695**									
Gender									
Male	85	66^a^	78^a^	17^a^	54^a^	25^a^	13^a^	15^a^	12^a^
Female	83	54	71	8	43	19	6	8	6
Age									
18–24	71^a^	59^ab^	71	25^a^	49^abc^	26^a^	23^a^	22^bc^	21^a^
25–34	85	64^b^	75	26^a^	56^c^	28^a^	24^a^	26^b^	21^a^
35–44	85^b^	65^b^	76	17	51^bc^	28^a^	12	18^c^	13
45–54	82^b^	57^ab^	75	7^b^	42^a^	16^b^	4^b^	5^a^	4
55–64	83^b^	58^ab^	74	2^c^	43^ab^	18^b^	2^bc^	2^a^	1^b^
65 +	87^b^	55^a^	72	4^bc^	48^a^	18^b^	1^c^	3^a^	0^b^
Income									
$0–$24,999	74^a^	56	66^a^	9^a^	37	14^a^	8^a^	10^ab^	6^a^
$25,000–$49,999	84^b^	57	73^b^	8^a^	46^a^	17^ab^	6^a^	8^a^	6^a^
$50,000–$74,999	88^b^	60	78^bc^	9^a^	52^ab^	21^bc^	7^a^	8^a^	7^ab^
$75,000–$99,999	88^b^	63	81^c^	16^b^	55^b^	28^cd^	13^b^	15^bc^	11^bc^
$100,000 and higher	87^b^	63	77^bc^	21^b^	58^b^	34^d^	16^b^	18^c^	15^c^
Region									
Northeast	91^a^	55^a^	72^b^	8^b^	48^a^	22^a^	6^b^	6	6^a^
South	82^bc^	62^b^	72^b^	14^a^	51	25^a^	12^a^	14^a^	11^b^
Midwest	84^b^	63^b^	79^a^	10^ab^	44^a^	16	8^ab^	11^a^	9^ab^
West	79^c^	55^a^	73^ab^	14^a^	48^a^	22^a^	10^a^	11^a^	7^a^
Raises or hunts for meat									
Yes	87	79^a^	84^a^	39^a^	68^a^	45^a^	40^a^	50^a^	37^a^
No	83	56	72	7	44	18	5	5	4

a, b, c *Percentages within the demographic category with different letters were significantly different (P < 0.05)*.

#### Gender

A higher percentage of men consumed all included species frequently with the exception of poultry.

#### Age

A lower percentage of the ages 18–24 (71%) consumed poultry frequently when compared to all other age groups. There were no clear trends in age for the frequent consumption of pork or fish, and there were no statistical differences in the percentages of respondents who consumed beef frequently for the different age groups. Lamb and buffalo were consumed frequently by higher percentages of respondents aged 18–24 (25, 23%) and 25–34 (26, 24%) when compared to those older than 45. Higher percentages of respondents aged 18–44 consumed shellfish and game species frequently when compared to those aged 45 and older.

#### Income

A lower percentage of respondents (74%) with an income of $0–24,999 consumed poultry frequently when compared to all other income categories. The percentage of respondents who consumed pork frequently did not differ statistically between the income groups. A lower percentage of respondents with an income of $0–24,999 consumed beef (66%) and fish (37%) frequently, and the percentage increased as the income level increased. Lower percentages of those with incomes of $0–74,999 consumed lamb and buffalo frequently when compared to the higher incomes. There was not a clear trend in the frequent consumption of game species as related to income.

#### Region

A higher percentage of respondents from the Northeast (91%) consumed poultry frequently when compared to the other regions of residence. Higher percentages of respondents from the South (62%) and the Midwest (63%) consumed pork frequently when compared to the Northeast or West. Higher percentages of respondents from the Midwest consumed beef frequently when compared to the Northeast or the South. Lower percentages of respondents from the Northeast consumed lamb when compared to the South and West. For fish, higher percentages of respondents from the South (51%) consumed it frequently when compared to all other regions. A lower percentage of respondents from the Midwest (16%) consumed shellfish frequently when compared to all other regions. The percentage of respondents who consumed buffalo frequently was higher for the South (12%) and West (10%) when compared to the Northeast. A lower percentage of respondents (6%) from the Northeast consumed game species frequently when compare to the other regions. A higher percentage of respondents from the South consumed exotics frequently (11%) when compared to the Northeast or the West.

#### Raises or Hunts for Meat

A higher percentage of respondents who indicated they raised or hunted for at least some of the meat they consumed, consumed all categories of meat frequently when compared to those who did not raise or hunt for meat.

### Meat Labels

When asked which labels respondents looked for when purchasing meat products, 39% of respondents indicated they looked for the USDA organic label and 38% indicated they looked for the Non Genetically Modified Organism (GMO) project label; the percentages of respondents who selected these labels were statistically higher than the percentage that selected the other labels Table 3. Ten percent of respondents selected the Animal Welfare Approved label and 8% of respondents selected the American Humane Certified label, statistically higher than the percentage of respondents who selected the remaining labels. The Certified Humane (17% of respondents) and Cruelty Free (16% of respondents) labels were the next most-selected, followed by the Certified Sustainable Seafood label (13% of respondents). Only 6% selected the Global Animal Partnership label. For use in later analysis, a variable indicating whether the respondents looked for at least one animal welfare associated label was created. Animal welfare associated labels included Animal Welfare Approved, Certified Humane, American Humane Certified, Global Animal Partnership, and Cruelty Free. Assuming those who do not consume meat would not look for any meat label when making a meat purchase, 35% of the total sample looked for an animal welfare related label. A statistically higher percentage of pet owners, 39%, looked for welfare labels when compared to the percentage of non-pet owners (25%).

**Table 3:** Labels^1^ respondents look for when buying meat products.

**Label**	**Percentage of respondents**	**Label**	**Percentage of respondents**
USDA Organic	39%^a^	American Humane Certified	8%^b^
Non-GMO Project Verified	38%^a^	Certified Sustainable Seafood MSC	13%^d^
Animal Welfare Approved	10%^b^	Global Animal Partnership	6%^e^
Certified Humane Raised & Handled	17%^c^	Cruelty-Free	16%^c^

Percentages of respondents who consume meat or have someone in the household that consumes meat (multiple selections permitted). N = 1,649.

1 Respondents were presented with images of the labels, but in this table descriptions of the labels are provided.

a, b, c *Percentages of respondents with different letters were significantly different (P < 0.05)*.

### Turkey Knowledge

When asked to identify a turkey/turkeys from pictures, 4% of respondents selected the chicken, 2% of respondents selected the duck, 94% of respondents selected the wild turkey, and 90% of respondents selected the commercial turkey (Table 4). Only 83% of respondents correctly selected the wild turkey and the commercial turkey without selecting the other non-turkey species. When considering combinations of selections, 9% of respondents correctly selected the wild turkey, but failed to select the commercial turkey. Four percent of respondents correctly selected the commercial turkey, but failed to select the wild turkey. Seventy-three percent of respondents correctly selected the wild turkey, and 10% of respondents selected the commercial turkey as the wild turkey (Table 4). Fifty-eight percent of respondents correctly identified the commercial female turkey, and 16% of respondents identified the wild female turkey as a commercial female turkey; the remaining respondents selected neither (Table 4).

**Table 4:** Respondent identification of pictures^a^ of turkeys (*N* = 1,695).

**Responses to the question: which of the following pictures is a/are turkeys?**			
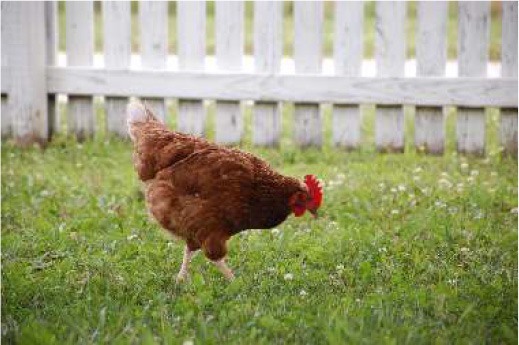	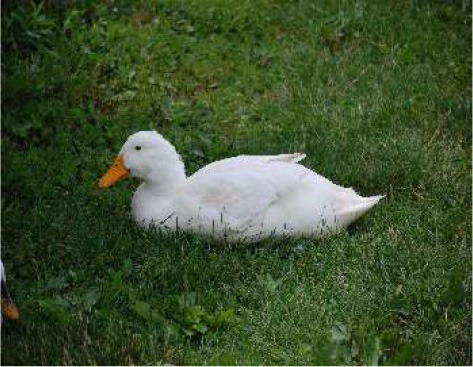	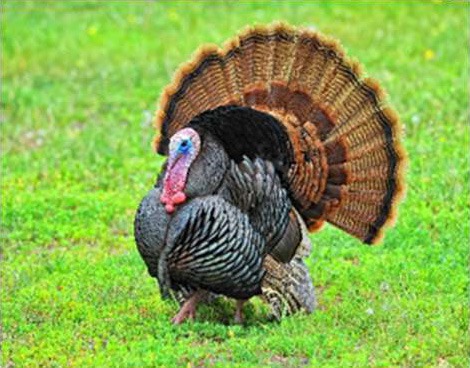	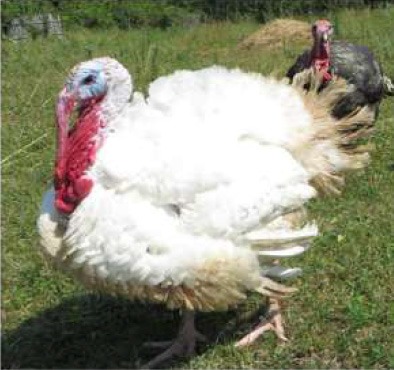
4% of respondents selected the chicken	2% of respondents selected the duck	94% of respondents selected the wild turkey	90% of respondents selected the commercial turkey
**Responses to the question: which of these pictures is a wild turkey?**			
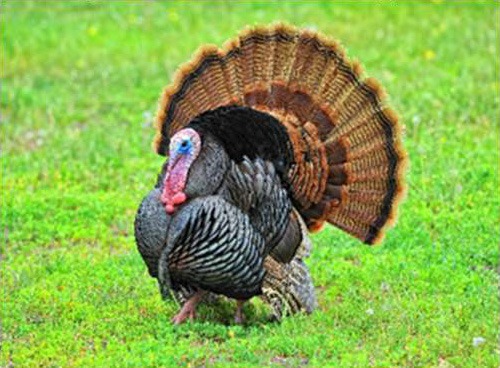	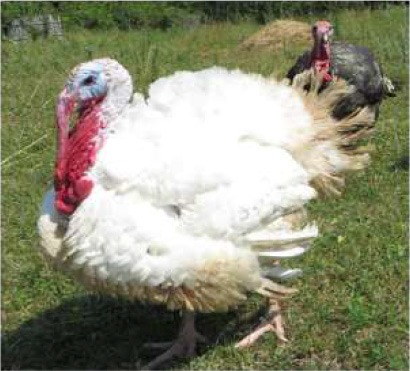		
73% of respondents selected the wild male turkey	10% of respondents selected the commercial male turkey		
**Responses to the question: which of these pictures is a female commercial farm turkey?**			
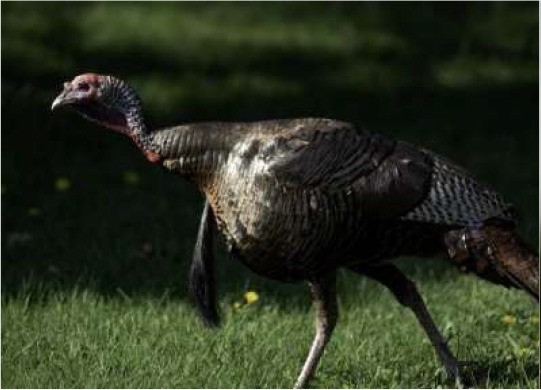	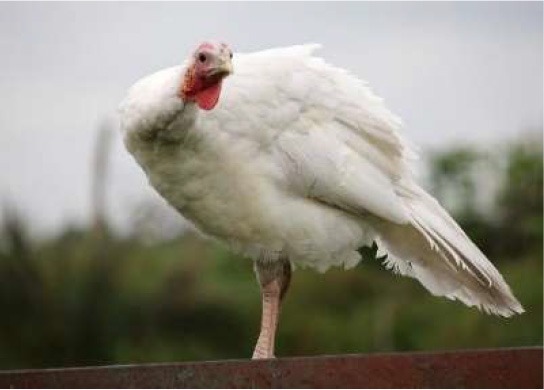		
16% of respondents selected the wild female turkey	58% of respondents selected the commercial female turkey		

a *Pictures were obtained from public domain sources or were the authors' personal photos, used with permission*.

Results pertaining to respondent's turkey knowledge are presented in Table 5. For the question “what is the average weight of a mature hen (female turkey),” 32% of respondents selected the correct answer (<25 pounds), 29% answered I don't know, and 21% answered about 25 pounds. For the question “how long do turkey eggs incubate before they hatch,” 76% selected I don't know, 9% selected 10–20 days, and 9% selected 20–25 days. Only 5% of respondents selected the correct answer (more than 25 days). When respondents were asked to indicate their level of knowledge of overall turkey production, 34% of respondents selected 1, and the mean score was 2.645. Selecting the correct live weight of a mature market hen (female) was not correlated with the self-reported knowledge of overall turkey production. However, selecting the correct length of time a turkey egg is incubated was positively correlated with the self-reported knowledge of overall turkey production (0.2495). Respondents who selected “I don't know” in response to the questions regarding commercial farmed hen weight (−0.2969) and length of egg incubation time (−0.4198) were negatively and statistically significantly correlated with the self-reported knowledge of overall turkey production.

Table 5Responses to turkey production questions (*N* = 1,695).

**Table:** 

**Answer choice**										**Percentage of respondents**
**What is the average weight of a mature market hen (female turkey)?**										
Less than 25 pounds^*^										32
About 25 pounds										21
About 30 pounds										12
About 35 pounds										4
More than 25 pounds										2
I don't know										29
**How long do turkey eggs incubate before hatching?**										
Less than 10 days										1
10–20 days										9
20–25 days										9
More than 25 days^*^										5
I don't know										76

**Table:** 

**Self-reported knowledge of overall turkey production on a scale of 1(no knowledge) to 7(very knowledgeable.)**										
Knowledge scale	1	2	3	4	5	6	7	Mean	SD
% of respondents	34%	22%	15%	15%	6%	4%	4%	2.645	1.697

**Table:** 

**Respondents' level of concern for the welfare of turkeys from 1 (very unconcerned) to 7 (very concerned)**.										
Turkey types^1^	1	2	3	4	5	6	7	Mean	SD	Rank
Concern for commercially farmed turkeys	8%	7%	10%	21%	19%	14%	21%	4.636^a^	1.827	1
Concern for turkeys in backyard flocks	12%	11%	14%	24%	16%	9%	14%	4.062^b^	1.844	2
Concern for wild turkeys	16%	12%	13%	21%	14%	10%	13%	3.875^c^	1.956	3

**Table:** 

**Respondents' rankings of concern for conditions that turkeys may face from 1 (most concerning) to 5 (least concerning)**.										
Conditions	1	2	3	4	5	Mean	SD	Rank
Illness	29%	26%	21%	13%	11%	2.508^a^	1.314	1
Poor nutrition	25%	32%	21%	13%	9%	2.471^a^	1.239	1
Housing type	26%	19%	23%	20%	12%	2.732	1.354	2
Hot or cold weather	13%	14%	23%	29%	21%	3.308	1.300	3
Transportation	7%	8%	13%	24%	48%	3.981	1.252	4
**Respondents' rankings of turkey welfare attributes from 1 (what they care about the most) 5 (what they care about the least)**										
Welfare attributes	1	2	3	4	5	Mean	SD	Rank
Space to move around	33%	29%	17%	13%	9%	2.366^a^	1.293	1
Veterinary health and wellness	29%	19%	21%	16%	14%	2.680^e^	1.414	2
Ability to perform natural behaviors	20%	24%	24%	19%	13%	2.812^c^	1.317	3
No feather loss or visible injuries	11%	17%	23%	26%	22%	3.304^d^	1.290	4
Decreased aggression between animals	7%	11%	15%	25%	42%	3.837^b^	1.265	5

* correct answer.

a, b, c Means of the two attributes were significantly different (P < 0.05).

1 *For all ranking/rating questions, answer choices were presented to respondents in random order*.

### Welfare of Turkeys and Other Animals

In response to the question “can animals feel pain,” 93% of respondents indicated they think animals can feel pain, 2% chose no, and 5% chose I don't know. Respondents were then specifically asked if turkeys could feel pain. Eighty-nine percent of respondents indicated that turkeys can feel pain. Four percent indicated turkeys cannot feel pain and 7% chose I don't know. Three percent of respondents indicated that animals could feel pain, but turkeys could not. There was no correlation between respondents' self-reported knowledge and whether they reported that turkeys could feel pain or not (*r* = −0.02, *P* = 0.34).

Respondents had the greatest concern for commercially farmed turkeys (4.636), followed by turkeys in backyard flocks (4.062), and then wild turkeys (3.875) (Table 5). Respondents were also asked to rank 6 conditions turkeys may face. The top concerns (statistically tied for first) at the mean level were poor nutrition (2.471) and illness (2.508). The other concerns in order were housing type (2.732), hot or cold weather (3.308), and transportation (3.981) (Table 5). Turkey welfare attributes that respondents cared the most about were ranked from most to least as: space to move around (2.366), followed by veterinary health and wellness (2.680), ability to perform natural behaviors (2.812), no feather loss or visible injuries (3.304), and decreased aggression between animals (3.837). For turkey housing systems, respondents ranked the picture of the birds outdoors 1st at the mean level (1.29), followed by the caged turkeys (2.316), and the birds in the curtain-sided barn (2.394) (Table 6).

**Table 6:** Ranking of housing conditions from 1 (best condition) to 3 (worst condition) (*N* = 1,695).

**Housing condition^**1**^**	**1**	**2**	**3**	**Mean**	**SD**	**Mean rank**
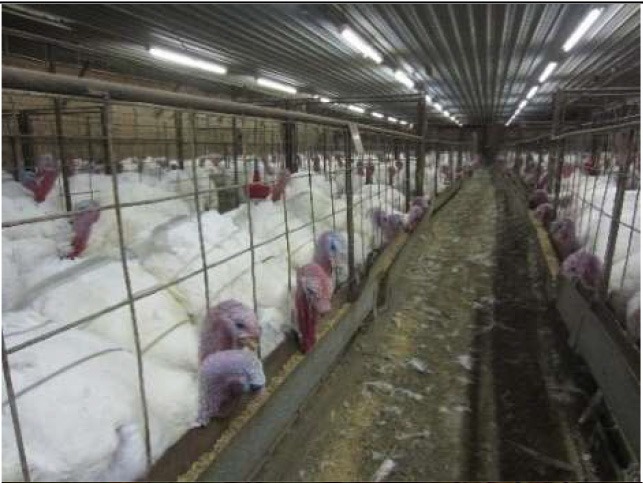	158	844	693	2.316^b^	0.634	2
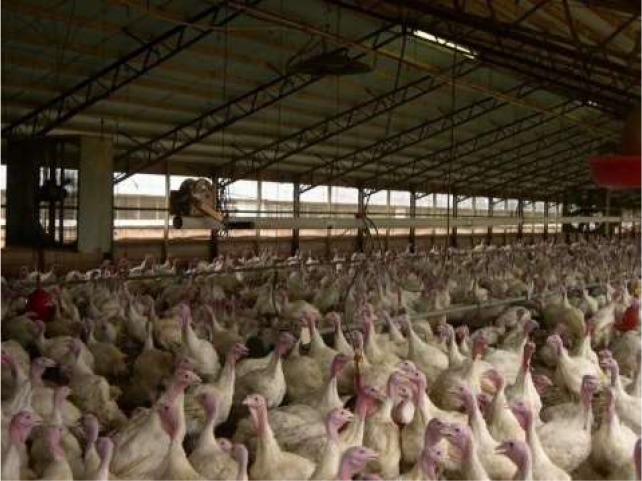	146	735	814	2.394^a^	0.641	3
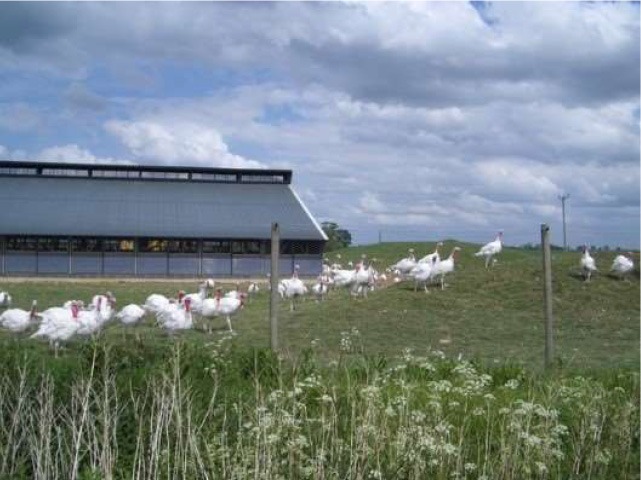	1,391	116	188	1.29^c^	0.654	1

1 Pictures were obtained from public domain sources.

a, b, c *All mean rankings were significantly different (P < 0.05)*.

To evaluate respondents' level of concern for turkeys relative to other species, they were presented with 7 unlabeled pictures of animals. For the purposes of discussion, the pictures will be referred to as they are labeled in Table 7, namely: crab, commercial turkey, wild turkey, chicken, beef cow, pig, and dairy cow. Respondents moved between 1 and 7 animals to one of the three buckets, and on average moved 6.6 animals to buckets. For all animals with the exception of the crab, a higher percentage of respondents were concerned about that animal's welfare when compared to neutral or not concerned. A higher percentage of respondents were concerned for the beef cow (59%), pig (59%), and dairy cow (60%) when compared to the crab, wild turkey, and chicken. A lower percentage of respondents were concerned for the wild turkey (48%) when compared to all other species with the exception of the crab. The lowest percentage of respondents (27%) were concerned for the welfare of the crab. Conversely, the highest percentage of respondents (35%) selected they were not concerned about the crab's welfare when compared to the other species.

**Table 7:** Level of concern regarding the welfare of the following animals^1^.

**Animal as seen by respondent**	**Concerned about this animal's welfare**	**Neutral about this animal's welfare**	**Not concerned about this animal's welfare**
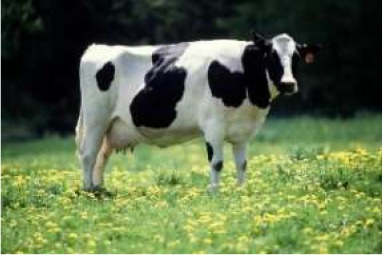 Dairy Cow^2^	60%^a^^Ψ^	22%^b^^Ψ^	13%^c^^Δ^
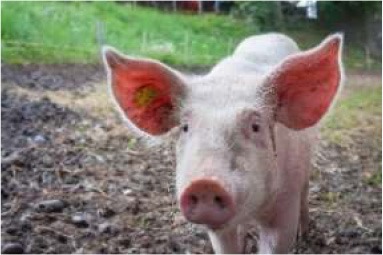 Pig	59%^a^^ΔΨ^	24%^b^^Ψ^	13%^c^^Δ^
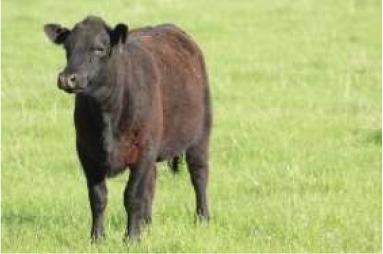 Beef Cow	59%^a^^ΔΨ^	23%^b^^Ψ^	13%^c^^Δ^
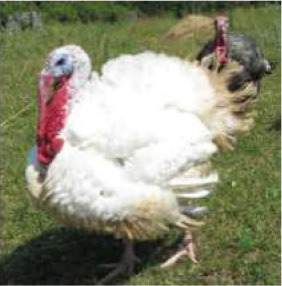 Commercial Turkey	56%^a^^ΔΓ^	28%^b^^ΔΓ^	12%^c^^Δ^
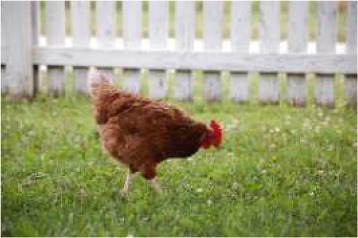 Chicken	55%^a^^Γ^	27%^b^^Γ^	14%^c^^Δ^
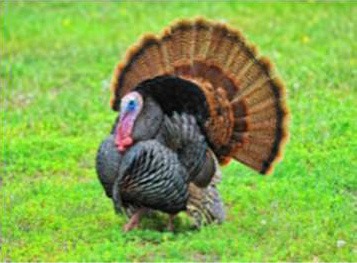 Wild Turkey	48%^a^^Θ^	31%^b^^Δ^	16%^c^
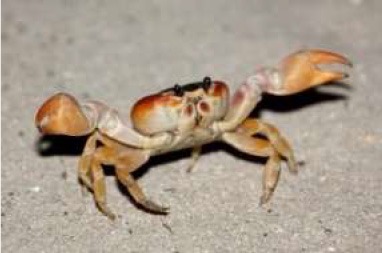 Crab	27%^Ω^	34%s^a^^Ω^	35%^a^^Γ^

1 Pictures were obtained from public domain sources or were the authors' personal photos, used with permission.

a, b, c Percentages of respondents with different lower case letters differed significantly across the row (P < 0.05).

Ψ, Δ, Γ, Θ, Ω *Percentages of respondents with different Greek letters differed significantly down the column (P <0.05). ^2^Respondents did not see picture labels*.

In the logit models, several factors affected respondents' concern for the seven animals (Table 8). Being female increased the probability of being concerned for the wellbeing of commercial turkeys (0.083), the chicken (0.077), the beef cow (0.092), the pig (0.087), and the dairy cow (0.109) when compared to being male. Having a pet increased the probability of being concerned for all animals with the exception of the crab. Working in agriculture decreased the probability of being concerned for the wild turkey (−0.271) when compared to those not working in agriculture. Having an income under $49,000 decreased the probability of being concerned for the welfare of the beef cow (−0.044) and the dairy cow (−0.051). Residence in the Northeast decreased the probability of being concerned for the welfare of the crab (−0.061) when compared to those from the West. Residence in the South decreased the probability of being concerned about the crab (−0.055), the commercial turkey (−0.058), the wild turkey (−0.054), and the chicken (−0.063) when compared to the West. Residence in the Midwest decreased the probability of being concerned about the welfare of the crab (−0.067) when compared to the West. Respondents who raised or hunted for at least some of the meat they consumed had a lower probability of being concerned for the welfare of all the animals with the exception of the crab and commercial turkey. Eating poultry frequently increased the probability of being concerned for the commercial turkey (0.068) and the chicken (0.054). Consuming pork frequently increased the probability of being concerned for the welfare of pigs (0.044). Consuming beef frequently decreased the probability of being concerned for dairy cows (−0.051).

**Table 8:** Logit models of respondents' concern for 7 animals.

		**Crab^**1**^**	**Commercial Turkey^**2**^**	**Wild Turkey^**3**^**	**Chicken^**4**^**	**Beef Cow^**5**^**	**Pig^**6**^**	**Dairy Cow^**7**^**
Female	Coefficient (SE)	0.170 (0.114)	0.346^***^ (0.102)	0.151 (0.101)	0.320^***^ (0.101)	0.391^***^ (0.103)	0.371^***^ (0.103)	0.471^***^ (0.103)
	Marginal E. (SE)	0.033 (0.022)	0.083^***^ (0.024)	0.037 (0.025)	0.077^***^ (0.024)	0.092^***^ (0.024)	0.087^***^ (0.024)	0.109^***^ (0.023)
Has a pet	Coefficient (SE)	0.118 (0.121)	0.400^***^ (0.107)	0.496^***^ (0.108)	0.302^**^ (0.107)	0.304^**^ (0.109)	0.364^***^ (0.108)	0.264^**^ (0.110)
	Marginal E. (SE)	0.023 (0.023)	0.096^***^ (0.025)	0.121^***^ (0.025)	0.073^**^ (0.026)	0.071^**^ (0.025)	0.0855^***^ (0.025)	0.061^**^ (0.025)
Works in AG	Coefficient (SE)	−0.400 (0.431)	−0.438 (0.349)	−1.114^**^ (0.410)	−0.711 (0.365)	−0.548 (0.350)	−0.561 (0.351)	−0.513 (0.348)
	Marginal E. (SE)	−0.078 (0.084)	−0.105 (0.084)	−0.271^**^ (0.099)	−0.171 (0.088)	−0.129 (0.082)	−0.132 (0.082)	−0.119 (0.080)
Income under $49,000	Coefficient (SE)	0.143 (0.113)	−0.042 (0.101)	−0.015 (0.100)	−0.071 (0.101)	−0.187^*^ (0.102)	−0.105 (0.102)	−0.219^**^ (0.103)
	Marginal E. (SE)	0.028 (0.022)	−0.010 (0.024)	−0.003 (0.024)	−0.017 (0.024)	−0.044^*^ (0.024)	−0.025 (0.024)	−0.051^**^ (0.024)
Northeast	Coefficient (SE)	−0.312^*^ (0.171)	0.024 (0.157)	−0.031 (0.156)	−0.012 (0.157)	0.086 (0.158)	0.222 (0.159)	0.141 (0.161)
	Marginal E. (SE)	−0.061^*^ (0.033)	0.006 (0.038)	−0.008 (0.038)	−0.003 (0.038)	0.020 (0.037)	0.052 (0.037)	0.033 (0.037)
South	Coefficient (SE)	−0.283^**^ (0.144)	−0.241^*^ (0.133)	−0.224^*^ (0.132)	−0.262^**^ (0.133)	−0.025 (0.134)	−0.095 (0.134)	−0.105 (0.135)
	Marginal E. (SE)	−0.055^**^ (0.028)	−0.058^*^ (0.032)	−0.054^*^ (0.032)	−0.063^**^ (0.032)	−0.006 (0.031)	−0.022 (0.031)	−0.024 (0.031)
Midwest	Coefficient (SE)	−0.345^**^ (0.163)	−0.009 (0.149)	−0.069 (0.147)	−0.123 (0.148)	0.038 (0.150)	0.041 (0.150)	0.043 (0.151)
	Marginal E. (SE)	−0.067** (0.032)	−0.002 (0.036)	0.017 (0.036)	−0.030 (0.036)	0.009 (0.035)	0.010 (0.035)	0.010 (0.035)
Raises or hunts	Coefficient (SE)	−0.062 (0.171)	−0.047 (0.148)	−0.303^**^ (0.148)	−0.480^***^ (0.148)	−0.43^8**^ (0.148)	−0.438^**^ (0.149)	−0.39^4**^ (0.148)
	Marginal E. (SE)	−0.012 (0.033)	−0.011 (0.035)	−0.074^**^ (0.036)	−0.116^***^ (0.035)	−0.103^**^ (0.034)	−0.103^**^ (0.035)	−0.091^**^ (0.034)
**Frequent consumer of the animal, i.e., pork for pig, poultry for chicken etc**.								
	Coefficient (SE)	0.116 (0.138)	0.285^*^ (0.135)	−0.142 (0.135)	0.222^*^ (0.135)	−0.050 (0.117)	0.188^*^ (0.105)	−0.218^*^ (0.119)
	Marginal E. (SE)	0.023 (0.027)	0.068^*^ (0.032)	−0.034 (0.033)	0.054^*^ (0.032)	−0.012 (0.027)	0.044^*^ (0.024)	−0.051^*^ (0.027)

* Indicates significance at the 0.10 level,

** indicates significance at the 0.05 level,

*** indicates significance at the <0.001 level.

1 Log Likelihood: −977.78525, Prob> chi^2^: 0.1937, Pseudo R^2^:0.0063;

2 Log likelihood: −1141.5771, Prob>chi^2^: 0.0000, Pseudo R^2^: 0.0182;

3 Log likelihood: −1152.2231, Prob>chi^2^: 0.000, Pseudo R^2^: 0.0185;

4 Log Likelihood: −1143.5385, Prob>chi^2^: 0.0000, Pseudo R^2^: 0.0203;

5 Log Likelihood: −1123.88, Prob>chi^2^: 0.0000, Pseudo R^2^:0.0188;

6 Log Likelihood: −1122.9844, Prob>chi^2^: 0.0000, Pseudo R^2^: 0.0215;

7 *Log Likelihood: −1110.3956, Prob>chi^2^: 0.0000, Pseudo R2: 0.0238*.

Results of respondent's self-reported feelings after watching video clips of turkeys are presented in Table 9. The percentage of respondents who selected each word differed between the two videos with the exception of the words ecstatic (1%), environmental impact (5%), heirloom (1%), and factory farming (15% for the pecking video and 13% for the calm video). Four percent of respondents selected at least one of the positive emotional attributes: happy, joyful, ecstatic, or pleasant for both videos. Conversely, 27% of respondents selected at least one of the negative emotional attributes: sad, angry, confused, anxious, or depressed for both videos. A higher percentage of respondents who indicated they had low knowledge of overall turkey production (selected a 3 or less on the scale) selected the negative emotional attributes for both videos (20%) when compared to the percentage of respondents who indicated higher knowledge of overall turkey production (selected a 5 or above on the scale), 3%. A higher percentage of respondents who indicated they had low knowledge of overall turkey production selected production oriented terms such as production location, production method, local, certified organic, factory farming, or big agriculture (12%) when compared to the percentage of respondents who indicated higher knowledge of overall turkey production (3%).

**Table 9:** Respondents' self-reported feelings after watching a short video clip of turkeys (*N* = 1,695).

**Feeling^**a**^**	**Aggressive video**	**Non-aggressive video**
Happy	3%^+^	11%
Joyful	2%^+^	5%
Ecstatic	1%	1%
Pleasant	3%^+^	18%
Angry	24%^+^	6%
Confused	17%^+^	12%
Anxious	23%^+^	6%
Depressed	18%^+^	9%
Sad	37%^+^	17%
Production location	6%^+^	9%
Production method	9%^+^	13%
Environmental impact	5%	5%
Local	4%^+^	7%
Factory farming	15%	13%
Big agriculture	7%^+^	5%
Certified organic	2%^+^	3%
Indifferent	12%^+^	20%
Quality	4%^+^	12%
Taste	2%^+^	3%
Support of local economy	3%^+^	5%
Heirloom	1%	1%
Freshness	3%^+^	7%
None	13%^+^	16%

+ The percentage of respondents who selected that feeling differed between the two videos (*P* < 0.05).

a Words were presented to respondents in a random order.

## Discussion

### Demographics and Meat Consumption

Consistent with Bir et al. (10) and Ochs et al. (11) who found that 88% and 86% of respondents indicated they were the primary shopper for their households, respectively, 87% of respondents in this study indicated they were the primary shopper of their household. Byrd et al. (12) found that 14% of respondents in their nationally representative survey hunted. This number is slightly higher than the 10% found in this study, but may be attributed to the wording of the question. In this survey, respondents were asked specifically if they hunted for some of the meat they consumed, while Byrd et al. (12) simply asked if they hunted, which does not necessarily imply the consumption of the hunted animals, which may account for the difference.

Species level meat consumption is often indirectly measured or inferred, for example through USDA statistics including availability (a measure of food available at the retail level), but is not measured at the individual household level. Availability of poultry has more than doubled since 1970, and the availability of poultry surpasses beef, pork, and fish/shellfish (13). In addition to greater availability, per capita consumption of poultry exceeded that of total red meat consumption in 2018 [109.5 lb. (retail weight) of total red meat vs. 110 lb. of poultry; (14)]. Consistent with these USDA statistics, higher percentages of respondents consumed poultry when compared to the other species studied for the frequencies of consumptions from daily to 2–3 times a week. When considering the frequently consumed meat species, for most cuts available, beef is more expensive followed by pork, then chicken (15). This may begin to explain the trend found in this analysis that respondents with incomes <$24,000 consumed beef less frequently, and that the percentage of frequent consumers increased with increases in income level.

### Meat Labels

When consumers make purchasing decisions, they use available cues about the quality of the product to make their decisions (16). Cues, such as labels, can convey information about how the animals were raised, including animal welfare-related attributes. In a nationally representative survey of U.S. consumers, Bir et al. (17) found that higher percentages of respondents with children and higher incomes always read the information on meat, egg, or milk product packaging when making a purchasing decision. Tonsor and Wolf (18) found that respondents were willing to support mandatory labeling that indicates the use of gestation crates (stalls) for pigs and laying hen cage type; both the use of crates for pigs and cages for hens have received a lot of attention due to the implications that they have for pig and hen welfare, respectively. Several factors including environmental concerns and demographic factors influence consumers' attitudes about animal-welfare related food labels (19, 20). In our study, a higher percentage of pet owners looked for animal welfare labels when compared to non-pet owners, which is consistent with previous literature reporting linkages between pet ownership and increased concern for animal welfare (21). Animal welfare labels were selected less frequently overall than the Non-GMO and USDA organic labels in this study, with higher percentages of respondents looking for the USDA organic label when purchasing meat products. According to Hughner et al. (22), organic farming has been increasing by 12% each year in the U.S. For poultry production in particular, concern over antibiotic use has driven the development of organic poultry production (23). Van Loo et al. (24) found that consumers were willing to pay a positive amount for organically labeled chicken breasts, but were willing to pay an even higher amount for the USDA organic label. In light of these previous studies, it is not surprising that a higher percentage of respondents in this study looked for the USDA organic label compared to other labels. There do not appear to be other studies that have specifically examined consumers' trust and awareness of the animal welfare-related labels examined in this study, so further work is needed to examine how awareness and knowledge of particular food labels influences the selection of these labels.

### Turkey Knowledge

Only a small percentage of U.S. citizens are involved in agriculture, and for those who are not in involved in agriculture, their understanding of how food is produced is limited (25). The majority of respondents in our study were unfamiliar with our turkey questions, with 76% stating that they did not know the length of incubation of a turkey egg. The overall mean score for self-reported knowledge of turkey production was low (2.4), consistent with the notion that the majority of U.S. consumers have a poor understanding of turkey production, even though 85% of respondents indicated that they purchased turkey products. This research provides an initial examination of U.S. consumers' knowledge of turkeys and due to the length of the survey, further questions about turkeys and their welfare were not asked. Therefore, a limitation of the current study was that more questions about specific turkey production and husbandry practices, that are more pertinent to turkey welfare, were not asked. Further research is needed to determine relationships among knowledge of specific production practices and perceptions of turkey welfare. There do not appear to be any other studies that have evaluated U.S. survey respondents' abilities to identify commercial and wild turkeys among other poultry species. Our results indicate that 6% of respondents in our study selected the picture of the chicken or the duck when asked to identify the commercial turkey, and 26% of respondents in our study could not distinguish between a wild and a commercial turkey. While the majority of households purchase turkey products (86%), purchasing the meat products does not necessarily indicate that consumers are knowledgeable about the practices used to bring that product to market. Further research will be useful in determining how the frequency of consumption of a particular species is related to knowledge of that species.

### Welfare of Turkeys and Other Animals

In addition to having a poor understanding of animal agriculture, the public also has a poor understanding of the welfare of production animals (26, 27). In terms of perceptions relating to turkey welfare, 4% of respondents stated that turkeys cannot feel pain, and 7% said they did not know whether they could feel pain or not. Another 3% of people who stated that animals can feel pain stated that turkeys cannot feel pain, indicating a discrepancy in views and concerns regarding different species of animals. The ability of an animal to suffer or feel emotions such as pain is a central tenet of animal welfare. There do not appear to be other studies that have examined public perceptions about turkeys' ability to feel pain. However, in a study examining Dutch respondents' perceptions of pig and fish welfare, 22.0% of respondents indicated they did not know whether pigs could feel pain and 36.6% of respondents indicating they did not know whether fish could feel pain (28). Based on these results, the authors concluded that respondents' perceptions of animal emotions, including pain, may not be predictive of their preference for the welfare in pig or fish farming (28). Previous research examining Australian consumer attitudes toward and knowledge of chicken production reported a relationship between empathy toward chickens and lack of knowledge; respondents with the lowest knowledge scores were accepting of inadequate stunning of chickens during slaughter (29). Other research has also confirmed the association between lack of knowledge and lack of concern for animals (26, 27). However, there was no significant correlation between self-reported knowledge of turkey production and indicating that turkeys can feel pain. It is possible that respondent's self-reported level of knowledge was lower than reported, or that respondents' beliefs about pain are different from empathy and other concerns about animal welfare. Further research is needed to specifically examine the relationship between turkey knowledge and beliefs about turkey welfare and pain.

In addition to asking about respondents' beliefs regarding pain, respondents were also asked to rank their level of concern for various animal species, including turkeys (shown in pictures). Respondents ranked images of beef cows, pigs, and dairy cows higher than images of chickens, wild turkeys, and crabs. These results are consistent with those of Byrd et al. (30) who found that concern and acceptable uses for different species of animals varied. Higher percentages of respondents had concern for bison and elk, followed by beef cattle, dairy cattle, deer, chickens, farmed pigs, farmed turkeys, wild turkeys, feral pigs, and catfish. Interestingly, there was a statistically lower percentage of respondents who were concerned for the chicken when compared to the pig, unlike the Byrd et al. (30) study. This study utilized pictures of animals instead of words. Perhaps visually considering the animal changed respondent perceptions. Additionally, pigs were a subject of news during this data collection due to swine flu outbreaks, which began in August of 2018, and might have had an effect on consumer concern (31). The lowest percentage of respondents were concerned for the crab, which is unsurprising considering that crabs are often cooked alive and considering that scientists are still debating whether fish and invertebrates experience pain the same way as vertebrate animals.

Being female increased the probability of being concerned for the commercial turkey, beef cow, pig, and dairy cow. Increased concern for animal welfare by female respondents was also found by Morgan et al. (32), Vanhonacker et al. (33), and McKendree et al. (21). Having a pet increased the probability of being concerned for all species studied except for the crab. This finding, alongside the increase among pet owners of reading animal welfare labels, further solidifies previous findings that those with pets have greater concern for animal welfare (21). Working in and around agriculture, as well as living in rural communities has been found to decrease concern for farmed animals (33). Surprisingly, working in agriculture only statistically significantly decreased the probability of being concerned for the wild turkey. However, being from the Northeast, where crabs and crab harvesting is celebrated (34), decreased the probability of being concerned for the crab, which may be associated with exposure to crab production. Unsurprisingly, being a hunter decreased the probability of being concerned for all species studied with the exception of the crab and commercial turkey. Conversely, being a frequent consumer of poultry increased the probability of being concerned for the commercial turkey and chicken, and being a heavy consumer of pork increased the probability of being concerned for the pig. Studies by De Backer and Hudders (35) and Morgan et al. (32) found that in general vegetarians were more concerned for animal welfare when compared to non-vegetarians; however, the relationship between specific species consumption and concern was not studied. The relationship between people's concern for animals and their desire to eat meat is complicated. Loughnan et al. (36) suggest that meat-eaters suppress their level of concern for animals because they do not want to hurt animals but do want to consume meat. While meat-eaters' general concern for animals is lower than vegetarians' concern for animals, it is not clear how meat eaters' consumption of particular species affects their concern for those species relative to other species. It may be possible that heavy consumers are more concerned for the species they consume than those species they do not consume in order to mitigate feelings associated with animal consumption, even though their concern is still lower than that of people who do not eat meat.

Respondents ranked their concern for commercially farmed turkeys as being higher than that of turkeys in backyard flocks and wild turkeys, which may partially support the idea that they are more concerned about the animals they consume, but further research is needed to establish the relationship between consumption of specific types of animals and concern for the welfare of those animals. There is no research available regarding consumers' perceptions or knowledge of turkey farming and housing practices, so it is difficult to speculate about the reasons for respondents' rankings. Poor nutrition and illness were ranked as being the most concerning of the conditions that turkeys face, whereas space to move around, followed by veterinary health and wellness were ranked as the items that respondents cared the most about. This study did not examine conditions affecting turkey welfare in relation to food safety and quality outcomes, but it is possible that respondents' perceptions of how conditions influence product safety and quality could influence the degree of importance respondents place on various conditions turkeys experience. For example, previous research determined that food safety concerns outweighed those of animal welfare concerns (37), and that meat consumers ranked fresh meat attributes such as quality, taste, freshness, free of hormones and healthiness as being generally most important, but there were differences due to socio-demographic factors such as gender and age (38). It is important to note that consumer interest in animal welfare will likely influence consumers' future meat consumption (39), and consumers may begin to perceive animal welfare as a component of product quality. We are not aware of other research that has examined public concern for specific turkey welfare outcomes or how welfare concerns relate to product safety and quality, so these results provide a starting point for further research into turkey welfare concerns.

To more specifically examine respondents' perceptions of turkeys, respondents were presented with three pictures of turkey housing conditions and ranked the outdoor housing system as representing the best condition. No explanations of the pictures were provided. Respondents may have associated the outdoor housing system with organic or free range production, but we did not specifically ask this question. More respondents chose the indoor housing where turkeys appeared behind a fence within an artificially lit barn as the next best option over the indoor, curtain-sided barn. In a recent study, Kühl et al. (40) used pictures of dairy cow husbandry systems to examine German respondents' acceptance of the husbandry systems. They concluded that perceived “naturalness” was the most important factor influencing whether indoor housing systems were regarded as acceptable, but that “naturalness” was not only restricted to cows being able to have access to sunlight and fresh air. Similarly, Busch et al. (41) examined how modifications to pictures of farmed pigs influenced people's perceptions of pig welfare. Their results indicated that aspects of the environment, such as whether pigs were on slatted or straw flooring, had a greater effect on their perceptions than aspects of the pigs themselves, such as the pig's facial expression and body language. Respondents rated pigs on straw as having higher welfare, even when the pig on straw was depicted as “unhappy”-looking compared to the pig on a slatted floor (41). These results may shed some light on interpreting the responses received in this study. While turkeys in the curtain-sided barn would have some natural daylight and fresh air, this may not be enough for this situation to be rated as higher compared to the other indoor situation where turkeys were kept under fluorescent lighting, especially when turkeys in the curtain-sided barn may be perceived as being more crowded and dirtier. Respondents indicated that they cared the most about space for turkeys to move around, which may partly explain why they ranked the picture of the curtain-sided barn as being worst. A limitation of the current study is that images had not been tested with a panel or test audience prior to the survey, so it cannot be determined which aspects of the pictures were most influential in affecting respondents' selections. Further research is needed to understand consumers' perceptions of turkey housing and husbandry practices.

In addition to asking respondents to rank images of turkeys' housing conditions, we also examined respondents' perceptions of videos of turkeys displaying species-typical behavior. The videos depicted turkeys in a research setting, and were not representative of conditions on commercial turkey farms. Videos were not narrated, so respondents were free to form their own interpretations. The videos were of white turkeys in similar environments (wood shavings covering the floor, feed and water in containers on a metal platform), but displaying different behavior. In one video clip, the turkeys were eating, drinking, sitting, standing, and walking. In the other video clip, two of the turkeys were displaying aggression (pecking at and chasing each other around the room) and at times causing other birds to move out of the way. Greater percentages of respondents selected terms associated with negative feelings to describe their feelings about the video in which turkeys were displaying aggressive behavior, whereas greater percentages of respondents selected positive terms when describing their feelings about the other video. In addition to more respondents selecting negative terms, a greater percentage of respondents also selected big agriculture, and fewer selected terms such as certified organic, quality, taste and freshness in relation to the aggressive video. Interestingly, the percentage of respondents' who selected the terms production location and production method differed between videos, even though the environmental conditions were the same in both videos. No explanations for these terms were provided, so their interpretation was left up to the respondents. Respondents' perceptions could therefore have been influenced by factors and information other than what we presented to them and it is difficult to explain why selection of production-related terms differed among videos when the environment that turkeys were housed in were the same for both videos. One explanation could be respondents' self-reported knowledge of turkey production. A greater proportion of respondents reporting low knowledge selected production oriented terms compared to respondents who reported that they had higher knowledge of turkey production. Results from other studies (42, 43) provide further insights into how people's perceptions are influenced by videos and corroborate some of our findings. In the study by Tonsor and Wolf (42), participants that watched a video of cows on pasture (“happy cows” video) perceived more conventional milk as coming from cows fed organic feed, having pasture access and having appropriate levels of well-being; whereas participants that watched a video of lame cows in mud (“unhappy cows” video) perceived lower amounts of conventional milk as coming from cows fed organic feed, having pasture access and having appropriate levels of well-being. Although we did not examine respondents' perceptions before and after watching our turkey videos, our results are comparable to those of Tonsor and Wolf (42), because our videos of turkeys displaying aggressive behavior, which is generally perceived as negative, were described with lower frequency using terms associated with organic production. In another study, Musto et al. (43) reported that participants expected milk to be more acceptable after watching a video of semi-natural living conditions and expected milk to be less acceptable after watching the video of the intensive living conditions (43). These results are consistent with the notion that consumers and the public have a generally more positive perception of alternative (e.g., organic and free range) farming systems than conventional systems.

Several studies have been conducted to understand consumers' motivations for purchasing organic food [reviewed in Hughner et al. (22), Hemmerling et al. (44)]. General themes that have emerged include perceptions that organic food is healthier, tastes better, is better for the environment, is better from a food safety perspective, provides better animal welfare and supports the local economy (22). Similarly, Hemmerling et al. (44) reported that the attributes that purchasers of organic food products view as most important are health protection, taste, environmental protection and the use of fewer chemicals or pesticides, followed by other attributes such as naturalness, animal welfare and quality. If the public generally views organic food as being healthier and providing better animal welfare, then this may explain why respondents in our study selected negative terms together with the term big agriculture when describing their feelings about the aggressive video, and positive feelings in conjunction with terms related to certified organic, taste, freshness, and quality when describing their feelings about the other video.

## Conclusion

The increasing public concern for animal welfare drives the need for factors affecting meat consumption and animal welfare to be better understood. Our results indicated that meat consumption of different species varied by region of residence, income level, gender, and age, and differed depending on whether respondents hunted for some or all of the meat they consumed. Poultry, beef, and pork were consumed frequently, with poultry being consumed most frequently on a daily basis and 2–3 times per week compared to other meat. A total of 35% of meat consumers reported looking for an animal-welfare related label, but the USDA organic label was the most frequently sought out meat label compared to other labels tested in this study, which is consistent with general trends in increasing organic food production.

In this study, 86% of respondents indicated that they purchase turkey products, 83% of respondents correctly identified a wild and a commercial turkey, and respondents' overall self-reported level of turkey production knowledge was low. Our results further reveal insights into how the public regards specific animal-welfare related concerns for turkeys. The majority of respondents (93%) indicated that animals can feel pain, and 89% indicated that turkeys, in particular, can feel pain. Perceptions about animals' abilities to feel pain, and therefore perceptions of animal sentience, influence how people treat animals. These perceptions also influence whether people are likely to find certain agricultural practices acceptable or not. Concern for the welfare of the commercial turkey ranked below that of the dairy cow, but did not differ from concern for the beef cow, pig, or chicken. In contrast, concern for the welfare of the wild turkey was ranked only higher than that of the crab, which was ranked the lowest of all species tested. Another factor that influenced animal welfare concerns was pet ownership. Being a pet owner was associated with greater levels of concern for the commercial turkey, wild turkey, chicken, beef cow, pig, and dairy cow, but not the crab. Pet owners also reported looking for animal-welfare related food labels more frequently compared to non-pet owners. These results are consistent with previous studies reporting greater concern for animal welfare generally among pet owners compared to non-pet owners.

Poor nutrition and illness were ranked as being the most concerning conditions that turkeys may face, while respondents indicated that they cared the most about adequate space for turkeys to move about, followed by turkeys' health. Further research is needed to identify specific instances of how knowledge of the production of turkey or other poultry affects perceptions of poultry welfare. Understanding the perceptions of animal production methods is particularly important as the public is increasingly influencing legislation pertaining to farm animal housing and management.

## Data Availability Statement

The raw data supporting the conclusions of this manuscript will be made available by the authors, without undue reservation, to any qualified researcher upon request.

## Ethics Statement

The studies involving human participants were reviewed and approved by Purdue University Institutional Review Board. Written informed consent for participation was not required for this study in accordance with the national legislation and the institutional requirements.

## Author Contributions

CB, MD, NW, SZ, and ME contributed to the development of survey questions, design of the study, and writing and editing of the manuscript.

### Conflict of Interest

The authors declare that the research was conducted in the absence of any commercial or financial relationships that could be construed as a potential conflict of interest.

## Footnotes

^1^Regions were defined according to the U.S. Census Bureau as follows: Northeast includes Connecticut, Maine, Massachusetts, New Hampshire, Rhode Island, Vermont, New Jersey, New York, and Pennsylvania; Midwest includes Indiana, Illinois, Michigan, Ohio, Wisconsin, Iowa, Kansas, Minnesota, Missouri, Nebraska, North Dakota, and South Dakota; South includes Delaware, District of Columbia, Florida, Georgia, Maryland, North Carolina, South Carolina, Virginia, West Virginia, Alabama, Kentucky, Mississippi, Tennessee, Arkansas, Louisiana, Oklahoma, and Texas; and the West includes Arizona, Colorado, Idaho, New Mexico, Montana, Utah, Nevada, and Wyoming, Alaska, California, Hawaii, Oregon, and Washington.
